# Robust older adults in primary care: factors associated with successful aging

**DOI:** 10.11606/s1518-8787.2020054001735

**Published:** 2020-03-30

**Authors:** Luciana Colares Maia, Thomaz de Figueiredo Braga Colares, Edgar Nunes de Moraes, Simone de Melo Costa, Antônio Prates Caldeira

**Affiliations:** I Universidade Estadual de Montes Claros Centro de Ciências Biológicas e da Saúde Departamento de Clínica Médica Montes ClarosMG Brasil Universidade Estadual de Montes Claros - Unimontes. Centro de Ciências Biológicas e da Saúde (CCBS). Departamento de Clínica Médica. Montes Claros, MG, Brasil; II Universidade Estadual de Montes Claros Centro Mais Vida Eny Faria de Oliveira Montes ClarosMG Brasil Universidade Estadual de Montes Claros - Unimontes. Centro Mais Vida Eny Faria de Oliveira (CRASI-EFO). Montes Claros, MG, Brasil; III Universidade Federal de Minas Gerais Faculdade de Medicina Departamento de Clínica Médica Belo HorizonteMG Brasil Universidade Federal de Minas Gerais. Faculdade de Medicina. Departamento de Clínica Médica. Belo Horizonte, MG, Brasil; IV Universidade Estadual de Montes Claros Centro de Ciências Biológicas e da Saúde Departamento de Odontologia Montes ClarosMG Brasil Universidade Estadual de Montes Claros - Unimontes. Centro de Ciências Biológicas e da Saúde (CCBS). Departamento de Odontologia. Montes Claros, MG, Brasil; V Universidade Estadual de Montes Claros Centro de Ciências Biológicas e da Saúde Departamento de Saúde da Mulher e da Criança Montes ClarosMG Brasil Universidade Estadual de Montes Claros - Unimontes. Centro de Ciências Biológicas e da Saúde (CCBS). Departamento de Saúde da Mulher e da Criança. Montes Claros, MG, Brasil

**Keywords:** Older Adults, Healthy Aging, Healthy Lifestyle, Protective Factors, Primary Health Care, Cross-Sectional Studies

## Abstract

**OBJECTIVE:**

To estimate the prevalence of robustness among older adults assisted in primary health care and identify factors in successful aging.

**METHODS:**

This is a cross-sectional study conducted with older adults in Northern Minas Gerais, Brazil. Two questionnaires were used for data collection: the Brazilian Older Americans Resources and Services Multidimensional Function Assessment Questionnaire (BOMFAQ) and the Clinical-Functional Vulnerability Index IVCF-20). The adjusted prevalence ratios were obtained by robust Poisson regression. Statistical analysis was performed for older adults in general (60 to 107 years) and stratified by age: from 60 to 79 years and 80 years or more.

**RESULTS:**

A total of 1,750 older adults aged 60 to 107 years participated; between them, 48.7% were robust. Older adults aged 60 to 79 years (n = 1,421) and 80 years or more (n = 329) had a prevalence of robustness of 55.4% and 19.3%, respectively. Some factors associated with successful aging were: positive self-perception of health, dancing habits, walking habits, absence of cognitive impairment, absence of depressive symptoms and polypathology, as well as daily life independence. After adjustment by age, the absence of polypathology and independence for activities of daily living stand out for robustness between 60 and 79 years; in those aged 80 years and over, independence for activities of daily living and dance practice presented greater strength of association.

**CONCLUSION:**

The prevalence of robust older adults in primary care is considered satisfactory for the older population in general but decreases with age and is associated with the absence of diseases and disabilities. These results denote the need to redesign the health care system, focusing on promoting and preventing clinical-functional vulnerability.

## INTRODUCTION

The 21st century is characterized by an important change in the global population pyramid, based on the significant growth of older people, both in developed and developing countries^1^. This demographic phenomenon brings profound epidemiological changes, which imply new challenges for health systems^2^. It is necessary to minimize the consequences of the aging process, seeking to keep older adults functionally independent for as long as possible^1,2,5^. Individual aging is not the only cause of functional decline but the main risk factor for the accumulation of chronic health conditions, which tend to decrease functionality and quality of life, besides generating more costs for health systems^6^.

The expression “successful aging” arose from the acknowledgment of the individual, heterogeneous and irreversible nature of the aging process^7,8^ and can be understood as the reduction in the functional reserve without, however, compromising the necessary function for the activities of daily living^2^. Healthy older adults are those capable of managing their own life and determining when, where and how their leisure activities, social life and work will occur, regardless of the presence of comorbidities, autonomously and independently^4^. Rowe and Kahn’s classic definition of successful aging determines objective biomedical criteria, based on the absence of diseases and disabilities, maintaining physical and cognitive capacity, and active engagement with life^9^.

In a broader conception, successful aging would be the vector resulting from the multidimensional interaction between physical and mental health, independence in daily life, social integration, family support and economic independence^1,7^. This perspective is adopted in the most recent health care guidelines for older adults of the Brazilian Ministry of Health^10^and the World Health Organization (WHO)^1^. In this expanded conception of aging, although most older adults have at least one chronic disease, not everyone is limited by it and many have normal lives, with control of their conditions and satisfaction with life^2,4^. Thus, well-being in old age, or health in an integral sense, derives from the balance between the dimensions of the functional capacity of the older person and their environment, without necessarily meaning the absence of problems^4,11^; thus it is important to recognize the vulnerability strata of the subjects^10,12,13^.

Brazilian scientific literature still demands further discussion on this theme. The expansion of the primary care network, through the Family Health Strategy (FHS) teams, as well as the increase of the older population, make it imperative to recognize successful aging and its associated factors for an effective promotion of health. Given this context, this study aimed to estimate the prevalence of robustness among older people assisted in primary health care and identify factors associated with successful aging.

## METHODOLOGY

This is a population-based cross-sectional survey conducted in a city in Northern Minas Gerais, Brazil. Data were collected in 2017, interviewing the older adults assisted in primary health care (PHC) in the urban area. This year, the municipality had assistance coverage by FHS teams greater than 80%.

The sample size was based on the population estimate, and the formula for infinite population was used, with prevalence of the outcome equal to 50%, sample error of 3% and confidence interval of 95% (95%CI). The sampling was complex by clusters: regional health centers and FHS teams. Considering the sampling process, the number was multiplied by a correction factor for the design effect (deff) equal to 1.5 plus 10% for eventual losses.

The team of interviewers, composed of nurses and medical students, was specially trained for data collection. In addition, a pilot study was carried out for final calibration of instruments and interviewers (data not included in the final analysis). Data were collected at home and in the morning, evening or night periods, on all days of the week. Older adults not at their homes on at least three visits, on different days and times, even after previous scheduling, were considered losses.

Two surveys were used: the Brazilian version of Older Americans Resources and Services Multidimensional Function Assessment Questionnaire (BOMFAQ)^4,14^ and the Clinical-Functional Vulnerability Index IVCF-20^12,13^. BOMFAQ is a multidimensional tool, adapted and validated in Brazil^4,14^. The IVCF-20 was used for the screening of probability of clinical-functional vulnerability, with a score between 0 and 40 points. It identifies the frail older adults with sum greater than or equal to 15 points, pre-frail with a value of 7 to 14 and robust with a score less than or equal to 6^12,13^. In this sense, the screening recognizes older adults with lower clinical-functional vulnerability, which are probably the most active and successfully aging. In this study, the IVCF-20 presenting low score (robust older adults) was taken as synonymous with successful aging. Thus, the IVCF-20score was dichotomized to compose the dependent variable: less than or equal to 6 for robust older adults and greater than or equal to 7 for non-robust older adults.

The independent variables were composed by the sociodemographic profile (sex, age group, education, marital status and family income in minimum wages at the time – R$ 937.00) while the determinants of successful aging were based on Rowe and Kahn’s traditional model^9^. This model, although criticized, still has influence and is widely used in the literature^2,3,15^. It encompasses the domains and variables evaluated in this study: social engagement (self-perception of health, reading habits, dance practice and loneliness), upkeep of physical and cognitive capacity (walking, sports practice, cognitive impairment measured by the Mini Examination of Mental State [MMSE] and depressive symptoms by the Short Psychiatric Evaluation Schedule [SPES]) and absence of diseases and disabilities (polypathology and functional independence evaluated through their activities of daily life [ADL]). All the information aforementioned was obtained from BOMFAQ and dichotomized. Polypathology was considered as five or more self-reported diseases. Total independence for ADL would be conducting basic and instrumental activities without compromises, investigated by BOMFAQ (bedtime, bathing, dressing, combing hair, cutting toenails, going to the bathroom in time, eating, going out driving, climbing a flight of stairs, walking near home, cleaning the house, medicating on time, shopping and preparing meals).

Data were processed by the IBM® SPSS® software version 22.0, and bivariate analyses were performed; followed by multiple analysis, by Poisson regression with robust variance for all variables associated with the event studied up to 20% (p < 0.20). The variables associated with successful aging up to the significance level of 5% (p < 0.05) were kept in the final model. The analysis was performed for all older adults in the study (60 to 107 years) and then for the strata between 60 to 79 years (young-old) and 80 years or more (long-lived older adults). Due to the cluster-based, complex sampling, weighting was used to estimate prevalence ratios and 95%CI.

The research was approved by the research ethics committee of the main institution of study, by opinion no. 1,628,652. Older adults participating in the study signed an informed consent form. The secrecy and confidentiality of the information collected was ensured.

## RESULTS

The study included 1,750 older adults, of whom 844 (48.7%) were considered “robust,” 548 (31.2%) “pre-frail” and the remaining 357 (20.1%) “frail.” Regarding the sociodemographic characteristics of the group, we found that most of the participants were women (63.5%), literate (89.0%), had a partner or spouse (54.2%) and received up to two minimum wages (63.5%). Older adults between 60 to 69 years (PR = 1.15; 95%CI 1.11–1.19) and 70 to 79 years (PR = 1.09; 95%CI 1.06–1.13) showed a higher prevalence of robustness when compared with those aged 80 years and over, as shown in Table 1. The Figure presents the characterization in percentages of clinical-functional vulnerability by the IVCF-20 of the 1,750 older adults classified as “robust” and “non-robust,” stratified by age.

**Table 1:** Association between sociodemographic variables and successful aging (Poisson regression) for older adults enrolled in primary health care in Montes Claros, MG, Brazil, 2017.

Sociodemographic variables	N = 1,750 older adults n (%a)	Robust older adult (IVCF-20 score ≤ 6)				Bivariate analysis		Multiple analysis	
		
		Yes		No		p	PR (95%CI)	p	PR (95%CI)
	
		n	%^a^	n	%^a^				
Sex						<0.001		0.070	
Female	1,111 (63.5)	477	43.2	633	56.8		1		1
Male	639 (36.5)	367	58.2	272	41.8		1.11 (1.07–1.14)		0.98 (0.95–1.00)
Age group						< 0.001		< 0.001	
80 years or older	329 (18.5)	63	19.3	266	80.7		1		1
70 to 79 years old	569 (32.5)	257	45.5	312	54.5		1.17 (1.13–1.21)		1.09 (1.06–1.13)
60 to 69 years old	852 (49.0)	524	61.8	327	38.2		1.31 (1.26–1.35)		1.15 (1.11–1.19)
Literate						< 0.001			
No	201 (11.0)	58	28.9	143	71.1		1	0.235	1
Yes	1,545 (89.0)	785	51.1	762	48.9		1.15 (1.10–1.20)		1.03 (0.99–1.06)
Marital status						< 0.001			
Without partner	803 (45.8)	327	41.0	476	59.0		1	0.978	1
With a partner	947 (54.2)	518	55.1	429	44.9		1.15 (1.10–1.21)		1.00 (0.97–1.03)
Household income						0.316			
> 2 MW	1,053 (63.5)	300	50.5	298	49.5		1	-	-
Up to 2 MW	568 (36.5)	500	47.9	553	52.1		1.02 (0.98–1.05)		

IVCF-20: Clinical-Functional Vulnerability Index; PR: prevalence ratio; 95%CI: 95% confidence interval; MW: minimum wages at the time

^a^ Percentage adjusted by the sample correction factor.

**Figure:**
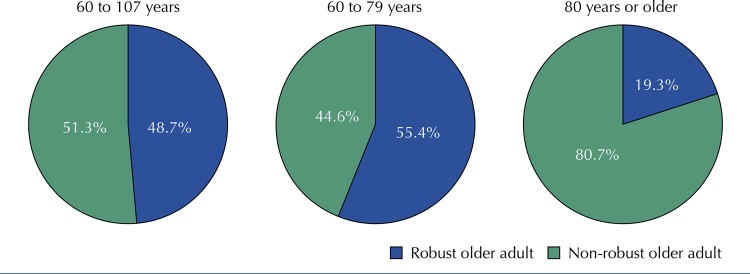
Characterization of clinical-functional vulnerability by the Clinical-Functional Vulnerability Index (IVCF-20) of older adults stratified by age (60 to 107 years, 60 to 79 years and 80 years or older) assisted in primary health care in Montes Claros, MG, Brazil, 2017.

Among the determinants of successful aging, in social engagement with life, 71.2% of the older adults had positive self-perception of life and 52.7% maintained reading habits. Regarding variables in upkeep of physical capacity and cognition, 28.5% had walking habits and 88.4% did not present cognitive impairment. Regarding the absence of diseases and disabilities, 27.7% did not present polypathology and 42.8% were totally independent for ADL. Robustness was associated to positive self-perception of health, dancing habits, absence of loneliness, walking habits, absence of cognitive impairment, absence of depressive symptoms, as well as not reporting five or more diseases (polypathology) and being independent for ADL (Table 2).

**Table 2:** Association between health-related variables and life habits and successful aging (Poisson regression) for older adults registered in primary health care in Montes Claros. MG. Brazil. 2017.

Variables	N = 1,750 older adults n (%^a^)	Robust older adult (IVCF-20 score ≤ 6) 60 to 107 years old				Bivariate analysis		Multiple analysis	
		
		Yes		No		p	PR (95%CI)	p	PR (95%CI)
	
		n	%^a^	n	%^a^				
Social engagement									

Self-perception of health						< 0.001		< 0.001	
Negative	511 (28.8)	105	20.7	406	79.3		1		1
Positive	1,239 (71.2)	739	60.0	499	40.0		1.48 (1.41–1.55)		1.19 (1.13–1.24)
Reading habits						< 0.001		0.690	
No	918 (52.7)	394	43.3	524	56.3		1		1
Yes	820 (47.3)	444	54.6	376	45.4		1.11 (1.05–1.16)		1.00 (0.97–1.05)
Dancing habits						< 0.001		< 0.001	
No	1,569 (90.2)	714	45.9	855	54.1		1		1
Yes	167 (9.8)	119	71.6	48	28.4		1.28 (1.19–1.38)		1.15 (1.09–1.27)
Loneliness						< 0.001		0.007	
Present	345 (19.8)	80	23.4	264	76.6		1		1
Absent	1,381 (80.2)	763	55.6	618	44.4		1.38 (1.31–1.41)		1.07 (1.02–1.13)

Upkeep of physical and cognitive capacity									

Walking habits						< 0.001		< 0.001	
No	1,241 (71.5)	501	40.7	740	59.3		1		1
Yes	494 (28.5)	334	68.3	160	32.4		1.31 (1.24–1.38)		1.13 (1.08–1.18)
Sports practice						0.003		0.959	
No	1,655 (95.4)	780	47.5	875	52.5		1		1
Yes	78 (4.6)	51	64.6	27	35.4		1.18 (1.06–1.32)		1.00 (0.91–1.09)
Cognitive impairment				< 0.001		< 0.001		< 0,001	
Present	201 (11.6)	42	21.7	159	78.3		1		1
Absent	1,545 (88.4)	801	52.2	744	47.8		1.34 (1.25–1.43)		1.18 (1.11–1.27)
Depressive symptoms				< 0.001		< 0.001		< 0,001	
Present	455 (25.9)	91	21.7	364	80.1		1		1
Absent	1,271 (74.1)	752	52.2	518	40.5		1.48 (1.41–1.55)		1.15 (1.10–1.21)

Absence of diseases and disabilities									

Polypathology						< 0.001		< 0.001	
Yes	489 (27.7)	67	13.7	422	86.3		1		1
No	1,260 (72.3)	777	62.1	483	37.9		1.67 (1.56–1.69)		1.33 (1.27–1.39)
Functional independence for activities of daily living						< 0.001	< 0.001	< 0,001	
No	998 (57.2)	287	29.1	711	70.9		1		1
Yes	751 (42.8)	557	74.8	194	25.2		1.56 (1.50–1.63)		1.30 (1.24–1.36)

IVCF-20: Clinical-Functional Vulnerability Index; PR: prevalence ratio; 95%CI: 95% confidence interval

^a^ Percentage adjusted by the sample correction factor.

In the group aged 60 to 79 years (n = 1,421), the prevalence of robustness was 55%, associated with the following variables: positive self-perception of health, dancing habits, absence of loneliness, walking habits, absence of cognitive impairment, absence of depressive symptoms, not reporting five or more diseases (polypathology) and being independent for ADL (Table 3). Among those aged 80 years or older (n = 329), the prevalence of robustness was 19.2%, associated with dance practice, walking, not having cognitive impairment, not reporting polypathology and total independence for ADL (Table 4).

**Table 3:** Association between health-related variables and life habits and successful aging (Poisson regression) for older adults between 60 and 79 years old registered in primary health care in Montes Claros. MG. Brazil. 2017

Variables	N = 1,421 older adults n (%^a^)	Robust older adult (IVCF-20 score ≤ 6) 60 to 79 years old				Bivariate analysis		Multiple analysis	
		
		Yes		No		p	PR (95%CI)	p	PR (95%CI)
	
		n	%^a^	n	%^a^				
Social engagement									

Self-perception of health						< 0.001		< 0.001	
Negative	406 (28.0)	97	23.9	309	76.1		1		1
Positive	1,015 (72.0)	684	67.6	330	32.4		1.32 (1.28–1.37)		1.14 (1.10–1.18)
Reading habits						< 0.001		0.679	
No	718 (51.0)	362	50.9	356	49.1		1		1
Yes	691 (49.0)	413	60.2	278	39.8		1.06 (1.02–1.10)		1.01 (0.92–1.04)
Dancing habits						< 0.001		< 0.001	
No	1,252 (88.9)	657	52.8	595	47.2		1		1
Yes	155 (11.1)	113	73.1	42	26.9		1.15 (1.09–1.23)		1.09 (1.03–1.14)
Loneliness						< 0.001		0.011	
Present	281 (19.8)	74	26.4	208	73.6		1		1
Absent	1,129 (80.2)	706	61.9	123	37.9		1.26 (1.22–1.31)		1.05 (1.01–1.09)

Upkeep of physical and cognitive capacity									

Walking habits						< 0.001		< 0.001	
No	961 (68.3)	458	47.9	503	52.1		1		1
Yes	446 (31.7)	314	71.0	132	29.0		1.18 (1.13–1.23)		1.07 (1.03–1.11)
Sports practice						< 0.018		0.846	
No	1,338 (95.2)	721	54.3	617	45.7		1		1
Yes	67 (4.8)	47	69.3	20	30.7		1.11 (1.02–1.21)		1.01 (0.94–1.07)
Cognitive impairment						< 0.001		0.005	
Present	109 (7.8)	37	34.4	72	65.6		1		1
Absent	1,310 (92.2)	743	57.1	567	42.9		1.15 (1.08–1.22)		1.08 (1.02–1.14)
Depressive symptoms						< 0.001		< 0.001	
Present	357 (24.8)	83	23.1	274	76.9		1		1
Absent	1,054 (75.2)	697	66.4	356	33.6		1.32 (1.28–1.37)		1.11 (1.06–1.15)

Absence of diseases and disabilities									

Polypathology						< 0.001		< 0.001	
Present	366 (25.5)	64	17.4	302	82.6				
Absent	1,054 (74.5)	717	68.4	337	31.6		1.38 (1.35–1.43)		1.21 (1.17–1.24)
Functional independence for activities of daily living						< 0.001		< 0.001	
No	743 (52.5)	260	35.3	483	64.7		1		1
Yes	677 (47.5)	521	77.4	156	22.6		1.33 (1.29–1.39)		1.18 (1.14–1.22)

IVCF-20: Clinical-Functional Vulnerability Index; PR: prevalence ratio; 95%CI: 95% confidence interval

^a^ Percentage adjusted by the sample correction factor.

**Table 4:** Association between health-related variables and life habits and successful aging (Poisson regression) for older adults over 80 years old registered in primary health care in Montes Claros. MG. Brazil. 2017.

Variables	N = 329 older adults n (%^a^)	Robust older adult (IVCF-20 score ≤ 6) 80 years or older				Bivariate analysis		Multiple analysis	
		
		Yes		No		p	PR (95%CI)	p	PR (95%CI)
	
		n	%^a^	n	%^a^				
Social engagement									

Self-perception of health						< 0.001		0.284	
Negative	105 (52.5)	08	8.3	97	91.7		1		1
Positive	224 (67.7)	55	24.5	169	75.5		1.10 (1.05–1.15)		1.04 (0.97–1.13)
Reading habits						0.135		0.828	
No	200 (60.4)	32	16.0	168	84.0		1		1
Yes	129 (39.6)	31	24.2	98	75.8		1.04 (0.99–1.10)		0.99 (0.91–1.07)
Dancing habits						0.045		0.035	
No	317 (96.4)	57	18.1	260	81.9		1		1
Yes	12 (3.6)	06	50.0	06	50.0		1.22 (1.02–1.48)		1.32 (1.02–1.71)
Loneliness						0.006		0.371	
Present	63 (19.7)	06	9.8	57	90.2		1		1
Absent	252 (80.3)	57	22.7	195	77.3		1.07 (1.02–1.13)		0.97 (0.89–1.04)

Upkeep of physical and cognitive capacity									

Walking habits						< 0.001		0.026	
No	280 (85.5)	43	15.5	237	84.5		1		1
Yes	48 (14.5)	20	41.9	28	58.2		1.17 (1.06–1.28)		1.16 (1.02–1.32)
Sports practice						0.243		-	
No	317 (96.4)	59	18.7	258	81.3		1		-
Yes	11 (3.6)	04	38.2	07	61.8		1.11 (0.93–1.34)		-
Cognitive impairment						< 0.001		0.004	
Present	92 (96.4)	59	18.7	258	81.3		1		1
Absent	11 (3.6)	04	38.2	07	61.8		1.10 (1.05–1.15)		1.11 (1.03–1.20)
Depressive symptoms						< 0.001		0.557	
Present	98 (30.5)	08	8.1	74.5	91.9		1		1
Absent	217 (69.5)	55	25.5	162	74.5		1.10 (1.05–1.15)		1.02 (0.95–1.01)

Absence of diseases and disabilities									

Polypathology						< 0.001		< 0.001	
Yes	123 (37.3)	03	2.7	120	97.3		1		1
No	206 (62.7)	60	29.1	146	70.9		1.16 (1.11–1.21)		1.24 (1.16–1.33)
Functional independence for activities of daily living						< 0.001		< 0.001	
No	255 (77.8)	27	10.7	228	89.3		1		1
Yes	74 (22.2)	36	49.5	38	50.5		1.26 (1.16–1.36)		1.33 (1.18–1.51)

IVCF-20: Clinical-Functional Vulnerability Index; PR: prevalence ratio; 95%CI: 95% confidence interval

^a^ Percentage adjusted by the sample correction factor.

## DISCUSSION

Among the older adults assisted by FHS teams in PHC, the prevalence of robustness can be considered satisfactory when evaluated among all the older population in the study. Approximately half of the older adults were stratified with low clinical-functional vulnerability, that is, potentially active and independent. Other studies presented a lower percentage of robust older adults, such as Hank^19^(8.5%), McLaughlin^15^(10.9%), Curcio^18^(24.4%), Canedo^2^(25%) and Bosch-Farre^20^(23.5% or 38.9%, according to instrument used). In the age-adjusted analysis, there was a prevalence of robustness almost three times higher among those aged 80 years or older, similar to observations of a study in Rio de Janeiro^2^. In the three analysis groups (all the older adults, 60 to 79 years and 80 years or more), the following variables were associated with robustness: dancing and walking habits, absence of cognitive impairment, not reporting polypathology and total independence for ADL.

However, it is important to consider the fact there is no standardization of instruments to measure successful aging. Similarly, categorization for age groups is different among studies, as well as the methodologies used. Rowe and Kahn’s classic proposal^9^, despite the scientific debate about it, continues to significantly influence all discussions on this subject^15^. Studies on the field are promising, but there is no conceptual consensus or universally standardized instruments for the evaluation^5,15,17,19^.

The aging process is challenging and requires innovative health care models, that is, capable of identifying and monitoring the clinical and functional conditions of the older population quickly, early and continuously, particularly in the public health network^1,5,10,11^. Currently, the health of older adults should be based on the interaction of the individual’s functionality (autonomy and independence) with their environment^1,5^. Thus, reflections on the positive, multidimensional and integrated evolution that constitutes the aging process begin in the literature^1,2,5,11,17,18,20^.

IVCF-20, used in this study, was developed for the stratification of clinical-functional risk and can be considered an indicator of good health conditions, health capacity or overall functionality^12,13^. It allows, in addition to classifying older adults with high and moderate functional vulnerability, to identify those considered of low clinical and functional risk, i.e. robust. Individuals identified with IVCF-20 lower than seven points are healthier, more active and should keep up with the usual follow-up focusing on health prevention and promotion measures on primary care^13^. Primary care is the gateway to the healthcare network and acts as a coordinator of care, and therefore needs to integrate other points of healthcare with greater complexity, according to the clinical and functional conditions of the older population^10,11^.

In this investigation, through the analysis of all older adults, age was the significant sociodemographic variable in the final model. The reduction in the prevalence of robustness among those aged 80 years or older was evidenced in this study. Other studies, despite using different instruments, but similar criteria, also showed that young-old adults are healthier and more robust^2,10,18,20^. However, aging includes multidimensional issues^7,8^ with involvement of different predictors, which are influenced in the course of life^1,11,16^. Younger and more independent older adults, in favorable environments, have better perception of life and are more active than long-lived ones^2^. In this study, not complaining about loneliness was associated with robustness in the group of all older adults and in those aged 60 to 79 years. Therefore, the interaction between functional independence and favorable environment promotes satisfaction and success in active lifelong engagement^1,2,21^. Studies with those aged 80 years or older are scarce and with limited methodologies, lacking research^2,22^ on social engagement.

Successful aging can be reproduced in functional capacity through physical and mental skills, both essential in autonomy and independence of each individual in a friendly (physical and social) environment. This is indispensable for the well-being of every human being, in the broadest sense, including domains such as happiness, satisfaction and self-efficacy^1,2^. In this investigation, older adults with positive self-perception of life as well as those with dancing habits presented less clinical-functional vulnerability, probably because they developed successful trajectories in aging, with particular attention to the variable of “dancing habits” associated with robustness between long-lived older adults (80 years or older) and young-old adults (60 to 79 years). The literature also showed that older adults capable of managing their own life (autonomy) and performing leisure activities revealed a self-perception of optimistic life, which contributes to a healthy and active old age^1,2,23^.

In addition to those with successful aging, we should emphasize our results regarding the prevalence of non-robustness, which affects especially long-lived older adults. Therefore, it is also necessary to invest in the training of health professionals regarding clinical-functional stratification and care centered on the particularities of pre-frail and frail older adults. Given this context, professional qualification of public health teams could contribute to recovering and rehabilitating strategies regarding functionality of vulnerable individuals. It is also important that public administrators provide structurally healthy environments for this population.

The intersectoral perspective of healthy and active aging, in friendly environments, can provide both maintenance and restoration of physical and cognitive capacity^1,11,21,27^. Moreover, the WHO, since 2007, through the Global Network for Age-friendly Cities, already recommends friendly environments for this population. The guide suggests adapting structures and integration between systems to promote successful and active aging^27^. Currently, the document *Brasil Amigo da Pessoa Idosa* (Age-Friendly Brazil) reinforces this previous proposal and makes commitments to municipalities that meet the requirements determined by the initiative^28^. This strategy, in accordance with the new epidemiological and social scenario of the Brazilian population, can collaborate to addressing the challenges regarding aging, causing impacts in a beneficial way in clinical and functional capacity.

Another significant point related to healthy longevity was the fact that the older population with cognitive and functionally independent abilities acquire healthy behaviors throughout life^2,24,26,29^ and can even enjoy digital technology in health management^30^. Such statement reiterates the findings of this research, in which the interviewees considered robust showed a higher prevalence of walking habits, as well as absence of cognitive impairment or depressive symptoms. Therefore, it is fundamental to establish strategies that keep the older population highly functional for as long as possible. This contributes to successful aging^20,31^, with lower morbidity and mortality rates^31^.

The absence of disabilities and diseases comprises another group of determining factors for successful aging^9^. In this research, older adults without reports of polypathology and with total independence for all ADL had superiority in clinical-functional capacity in relation to their peers. These data were also found for age-stratified analysis. Other studies have also shown how the presence of disabilities and polypathology produces clinical and functional vulnerability in individuals, with negative impacts in health and lifespan^1,2,4,10,18,31^.

Our results should be considered in the light of some limitations. The cross-sectional study made it impossible to determine causality. Data were reported by the older adults in question, and memory bias should be considered. In addition, data collection instruments have limitations, although they allow individuals to stratify their health characteristics. From this perspective, the importance of distinguishing and referencing “frail” older adults for multidimensional clinical evaluation and preparation of the care plan should be considered, at the secondary level of the public care network, with their counter-reference longitudinal follow-up by the family health team. Individuals in frail conditions and robust individuals continue with the care of PHC professionals trained in the particularities of the health of older population, according to manuals and/or health care guidelines.

Despite the limitations presented, the sample design and the high number of older adults included ensures representativeness of the group studied. The IVCF-20 instrument is a screening questionnaire, which allows the clinical-functional stratification of the older population. It is validated and easy to apply, and can be used by any health professional, facilitating the initial screening and monitoring of this population by FHS.

In summary, this study highlighted an important prevalence of active and healthy (robust) older adults, that is, those with low clinical and functional vulnerability. However, adjusted analysis for long-lived older adults showed a significant reduction in this prevalence, a result that reinforces the urgency to redesign health care systems for the older population, with a special focus on the particularities of different age groups, in order to prolong lifetime with active engagement and free of physical or cognitive disabilities. Therefore, the need to qualify professionals in the care of older adults, with health promotion and prevention of clinical-functional vulnerability, is emphasized, delaying the development of diseases and their complications, in addition to training of the PHC team for health recovery actions and rehabilitation of functionality.

In this context, many challenges exist. New research on this theme is recommended to stimulate the study of the relationship between determinants of successful aging and older adults with low clinical-functional vulnerability (robustness), as well as evaluations on planning and implementation of public policies for that population quota.

## References

[1] 1. World Health Organization. World report on ageing and health. Geneva: WHO; 2015 [citado 10 de fev. 2019. Disponível em: http://www.who.int/ageing/events/world-report-2015-launch/en/

[2] 2. Canêdo AC, Lopes CS, Lourenço RA. Prevalence of and factors associated with successful aging in Brazilian older adults: frailty in Brazilian older people Study (FIBRA RJ). Geriatr Gerontol Int. 2018;18(8):1280-5. 10.1111/ggi.13334 29717801

[3] 3. Jacob Filho W. Fatores determinantes do envelhecimento saudável. BIS Bol Inst Saude. 2009 [citado 10 de fev. 2019];(47):27-32. Disponível em: http://periodicos.ses.sp.bvs.br/scielo.php?script=sci_arttext&pid=S1518-18122009000200007&lng=pt.1-

[4] 4. Ramos LR. Fatores determinantes do envelhecimento saudável em idosos residentes em centro urbano: Projeto Epidoso, São Paulo. Cad Saude Publica. 2003;19(3):793-7. 10.1590/S0102-311X2003000300011 12806481

[5] 5. Beard JR, Officer A, Carvalho IA, Sadana R, Pot AM, Michel JP, et al. The World Report on Ageing and Health: a policy framework for healthy ageing. Lancet. 2016;387(10033):2145-54. 10.1016/S0140-6736(15)00516-4 PMC4848186 26520231

[6] 6. Moraes EN. The frail elderly and integral health management centered on the individual and the family. Rev Bras Geriatr Gerontol. 2017;20(3):307-8. 10.1590/1981-22562017020.170061

[7] 7. Depp CA, Jeste DV. Definitions and predictors of successful aging: a comprehensive review of larger quantitative studies. Am J Geriatr Psychiatry. 2006;14(1):6-20. 10.1097/01.JGP.0000192501.03069.bc 16407577

[8] 8. Bowling A, Iliffe S. Which model of successful ageing should be used? Baseline findings from a British longitudinal survey of ageing. Age Ageing. 2006;35(6):607-14. 10.1093/ageing/afl100 16951427

[9] 9. Rowe JW, Kahn RL. Successful aging. Gerontologist. 1997;37(4):433-40. 10.1093/geront/37.4.433 9279031

[10] 10. Ministério da Saúde (BR), Secretarira de Atenção à Saúde, Departamento de Ações Programáticas e Estratégicas, Coordenação de Saúde da Pessoa Idosa. Orientações técnicas para a implementação de linha de cuidado para atenção integral à saúde da pessoa idosa: no Sistema Único de Saúde - SUS. Brasília (DF); 2018. [citado 10 de fev 2019]. Disponível em: http://portalarquivos2.saude.gov.br/images/pdf/2017/novembro/13/Linha-cuidado-VERSAO-CONSULTA-PUBLICA-07nov2017.pdf

[11] 11. Veras RP, Oliveira M. Envelhecer no Brasil: a construção de um modelo de cuidado. Cienc Saude Coletiva. 2018;23(6):1929-36. 10.1590/1413-81232018236.04722018 29972500

[12] 12. Moraes EN, Carmo JA, Moraes FL, Azevedo RS, Machado CJ, Montilla DER. Índice de Vulnerabilidade Clínico Funcional-20 (IVCF-20): reconhecimento rápido do idoso frágil. Rev Saude Publica. 2016;50:81. 10.1590/s1518-8787.2016050006963 PMC5152846 28099667

[13] 13. Faller JW, Pereira DN, Souza S, Nampo FK, Orlandi FS, Matumoto S. Instruments for the detection of frailty syndrome in older adults: a systematic review. PLoS One. 2019;14(4):e0216166. 10.1371/journal.pone.0216166 PMC6488093 31034516

[14] 14. Blay SL, Ramos LR, Mari JJ. Validity of a Brazilian version of the Older Americans Resources and Services (OARS) mental health screening questionnaire. J Am Geriatr Soc.1988;36(8) 687-92. 10.1111/j.1532-5415.1988.tb07169.x 3403873

[15] 15. McLaughlin SJ, Connell CM, Heeringa SG, Li LW, Roberts JS. Successful aging in the United States: prevalence estimates from a national sample of older adults. J Gerontol B Psychol Sci Soc Sci. 2010;65B(2):216-26. https://doi:10.1093/geronb/gbp101 10.1093/geronb/gbp101 PMC2981444 20008481

[16] 16. Stowe JD, Cooney TM. Examining Rowe and Kahn’s concept of successful aging: importance of taking a life course perspective. Gerontologist. 2015;55(1):43-50. https://doi:10.1093/geront/gnu055 10.1093/geront/gnu055 PMC4986588 24906516

[17] 17. Whitley E, Popham F, Benzeval M. Comparison of the Rowe–Kahn model of successful aging with self-rated health and life satisfaction: the West of Scotland Twenty-07 Prospective Cohort Study. Gerontologist. 2016;56(6):1082-92. https://doi:10.1093/geront/gnv054 10.1093/geront/gnv054 PMC5181386 26970606

[18] 18. Curcio CL, Pineda A, Quintero P, Rojas Á, Muñoz S, Gómez F. Successful Aging in Colombia: the role of disease. Gerontol Geriatr Med. 2018;4:1-11. 10.1177/2333721418804052 PMC6207973 30397638

[19] 19. Hank K. How “successful” do older Europeans age? Findings from SHARE. J Gerontol B Psychol Sci Soc Sci. 2011;66(2):230-6. 10.1093/geronb/gbq089 PMC3041975 21135069

[20] 20. Bosch-Farré C, Garre-Olmo J, Bonmatí-Tomàs A, Malagón-Aguilera MC, Gelabert-Vilella S, Fuentes-Pumarola C, et al. Prevalence and related factors of Active and Healthy Ageing in Europe according to two models: results from the Survey of Health, Ageing and Retirement in Europe (SHARE). PLoS One. 2018;13(10):e0206353. 10.1371/journal.pone.0206353 PMC6205806 30372472

[21] 21. Navarro JHN, Andrade FP, Paiva TS, Silva DO, Gessinger CF, Bós AJG. Percepção dos idosos jovens e longevos gaúchos quanto aos espaços públicos em que vivem. Cienc Saude Coletiva. 2015;20(2):461-70. 10.1590/1413-81232015202.03712014 25715140

[22] 22. Knappe MFL, Espírito Santo ACG, Leal MCC, Marques APO. Envelhecimento bem sucedido em idosos longevos: uma revisão integrativa. Geriatr Gerontol Aging. 2015;9(2) 66-70.

[23] 23. Tavares RE, Jesus MCP, Machado DR, Braga VAS, Tocantins FR, Merighi MAB. Healthy aging from the perspective of the elderly: an integrative review. Rev Bras Geriatr Gerontol. 2017;20(6):878-89. 10.1590/1981-22562017020.170091

[24] 24. Valer DB, Bierhals CCBK, Aires M, Paskulin LMG. The significance of healthy aging for older persons who participated in health education groups. Rev Bras Geriatr Gerontol. 2015;18(4):809-19. 10.1590/1809-9823.2015.14042

[25] 25. Sixsmith J, Sixsmith A, Fänge AM, Naumann D, Kucsera C, Tomsone S, et al. Healthy ageing and home: the perspectives of very old people in five European countries. Soc Sci Med. 2014;106:1-9. 10.1016/j.socscimed.2014.01.006 24524960

[26] 26. Hicks SA, Siedlecki KL. Leisure activity engagement and positive affect partially mediate the relationship between positive views on aging and physical health. J Gerontol B Psychol Sci Soc Sci. 2017;72(2):259-67. 10.1093/geronb/gbw049 27162228

[27] 27. Organização Mundial da Saúde. Guia global: cidade amiga do idoso, 2007. Lisboa: Fundação Calouste Gulbenkian; 2009 [citado 10 de fev. 2019]. Disponível em: https://apps.who.int/iris/bitstream/handle/10665/43755/9789899556867_por.pdf?sequence=3

[28] 28. Ministério do Desenvolvimento Social. Estratégia Brasil Amigo da Pessoa Idosa: documento técnico. Brasília (DF); 2018 [citado 10 de fev. 2019]. Disponível em: https://www.mds.gov.br/webarquivos/publicacao/Brasil_Amigo_Pesso_Idosa/Documento_Tecnico_Brasil_Amigo_Pessoa_Idosa.pdf

[29] 29. Kollia N, Caballero FF, Sanchez-Niubó A, Tyrovolas S, Ayuso-Mateos JL, Haro JM, et al. Social determinants, health status and 10-year mortality among 10,906 older adults from the English Longitudinal Study of Aging: the ATHLOS Project. BMC Public Health. 2018;18(1):1357. 10.1186/s12889-018-6288-6 PMC6288914 30526556

[30] 30. Seçkin G, Hughes S, Yeatts D, Degreve T. Digital pathways to positive health perceptions: does age moderate the relationship between medical satisfaction and positive health perceptions among middle-aged and older internet users? Innov Aging. 2019;3(1):igy039. 10.1093/geroni/igy039 PMC6328706 30648160

[31] 31. Aliaga-Diaz E, Cuba-Fuentes S, Mar-Meza M. Promoción de la salud y prevención de las enfermedades para un envejecimiento activo y con calidad de vida. Rev Peru Med Exp Salud Publica. 2016;33(2):311-20. 10.17843/rpmesp.2016.332.2143 27656932

