# Supplementary material for: Robust older adults in primary care: factors associated with successful aging
Source: Rev Saude Publica. 2020 Mar 30;54:35. doi: 10.11606/s1518-8787.2020054001735 (PMC7112742; doi:10.11606/s1518-8787.2020054001735)
Supplement: Translation

# Translation: Idosos robustos na atenção primária: fatores associados ao envelhecimento bem-sucedido

**Authors:** Luciana Colares Maia, Thomaz de Figueiredo Braga Colares, Edgar Nunes de Moraes, Simone de Melo Costa, Antônio Prates Caldeira

**Affiliations:** I Brasil Universidade Estadual de Montes Claros - Unimontes. Centro de Ciências Biológicas e da Saúde (CCBS). Departamento de Clínica Médica. Montes Claros, MG, Brasil; II Brasil Universidade Estadual de Montes Claros - Unimontes. Centro Mais Vida Eny Faria de Oliveira (CRASI-EFO). Montes Claros, MG, Brasil; III Brasil Universidade Federal de Minas Gerais. Faculdade de Medicina. Departamento de Clínica Médica. Belo Horizonte, MG, Brasil; IV Brasil Universidade Estadual de Montes Claros - Unimontes. Centro de Ciências Biológicas e da Saúde (CCBS). Departamento de Odontologia. Montes Claros, MG, Brasil; V Brasil Universidade Estadual de Montes Claros - Unimontes. Centro de Ciências Biológicas e da Saúde (CCBS). Departamento de Saúde da Mulher e da Criança. Montes Claros, MG, Brasil

**Keywords:** Idoso, Envelhecimento Saudável, Estilo de Vida Saudável, Fatores de Proteção, Atenção Primária à Saúde, Estudos Transversais

## Abstract

**OBJETIVO:**

Estimar a prevalência de robustez entre idosos assistidos na atenção primária à saúde e identificar fatores de envelhecimento bem-sucedido.

**MÉTODOS:**

Trata-se de pesquisa transversal, realizada com idosos no norte de Minas Gerais, Brasil. Foram utilizados dois questionários para coleta de dados: *Brazilian Older Americans Resources and Services Multidimensional Function Assessment Questionnaire* (BOMFAQ) e Índice de Vulnerabilidade Clínico-Funcional (IVCF-20). As razões de prevalências ajustadas foram obtidas por análise de regressão de Poisson múltipla com variância robusta. A análise estatística foi realizada para os idosos em geral (60 a 107 anos) e estratificada por idade: de 60 a 79 anos e 80 anos ou mais.

**RESULTADOS:**

Participaram 1.750 idosos, com idade de 60 a 107 anos, sendo 48,7% robustos. Idosos de 60 a 79 anos (n = 1.421) e 80 anos ou mais (n = 329) apresentaram prevalência de robustez de 55,4% e 19,3%, respectivamente. Associaram-se ao envelhecimento bem-sucedido: autopercepção positiva da saúde, dançar, fazer caminhada, não ter comprometimento cognitivo, ausência de sintomas depressivos e de polipatologia, além de independência para atividades de vida diária. Após ajuste por idade, destacam-se para robustez entre 60 a 79 anos a ausência de polipatologia e a independência para atividades de vida diária; naqueles com 80 anos e mais, a independência para atividades de vida diária e a prática de dança apresentaram maior força de associação.

**CONCLUSÃO:**

A prevalência de idosos robustos na atenção primária pode ser considerada satisfatória para os idosos em geral, mas reduz com a idade e se associa com a ausência de doenças e incapacidades. Esses resultados denotam a necessidade de redesenhar o sistema de atenção à saúde, com foco na promoção e prevenção da vulnerabilidade clínico-funcional.

## INTRODUÇÃO

O século XXI caracteriza-se por importante alteração na pirâmide etária mundial, a partir do aumento expressivo de idosos, tanto nos países desenvolvidos quanto naqueles em desenvolvimento^1^. Esse fenômeno demográfico traz profundas mudanças epidemiológicos, que implicam novos desafios para os sistemas de saúde^2^. É preciso minimizar as consequências do processo de envelhecimento, buscando manter os idosos independentes funcionalmente, pelo maior período possível^1,2,5^. O envelhecimento individual não é causa de declínio funcional por si só, mas representa o principal fator de risco para o acúmulo de condições crônicas de saúde, que tendem a diminuir a funcionalidade e a qualidade de vida, além de gerar mais custos para o sistema de saúde^6^.

A expressão “envelhecimento bem-sucedido” surgiu a partir do entendimento da trajetória individual, heterogênea e irreversível no processo do envelhecer^7,8^ e pode ser entendido como a redução da reserva funcional sem, no entanto, comprometer a função necessária para as atividades do cotidiano^2^. O idoso saudável é aquele capaz de gerir sua própria vida e determinar quando, onde e como se darão suas atividades de lazer, convívio social e trabalho, independentemente da presença ou ausência de comorbidades, com autonomia e independência^4^. A definição clássica de Rowe e Kahn sobre o envelhecimento bem-sucedido determina critérios biomédicos objetivos, com base na ausência de doenças e incapacidades, na manutenção da capacidade física e cognitiva e no engajamento ativo com a vida^9^.

Em concepção mais ampla, o envelhecimento bem-sucedido seria o vetor resultante da interação multidimensional entre saúde física, saúde mental, independência na vida diária, integração social, suporte familiar e independência econômica^1,7^. Essa perspectiva é assumida nas mais recentes orientações da linha de cuidado para a saúde do idoso do Ministério da Saúde no Brasil^10^e da Organização Mundial de Saúde (OMS)^1^. Nessa concepção ampliada de envelhecer, embora a grande maioria dos idosos seja portadora de pelo menos uma doença crônica, nem todos ficam limitados por ela e muitos têm uma vida normal, com as enfermidades controladas e expressando a sua satisfação com a vida^2,4^. Desta forma, o bem-estar na velhice, ou a saúde num sentido integral, deriva do equilíbrio entre as dimensões da capacidade funcional do idoso e seu ambiente, sem necessariamente significar a ausência de problemas nas dimensões avaliadas^4,11^, sendo importante reconhecer os estratos de vulnerabilidade dos sujeitos^10,12,13^.

A literatura brasileira ainda demanda discussões científicas sobre essa temática. A ampliação da rede de atenção primária, por meio das equipes da Estratégia Saúde da Família (ESF), juntamente com o aumento da população idosa, tornam imperativos o reconhecimento da condição de envelhecimento bem-sucedido e de seus fatores associados para uma efetiva promoção de saúde. Nesse contexto, este trabalho objetivou estimar a prevalência de robustez entre idosos assistidos na atenção primária à saúde e identificar fatores associados ao envelhecimento bem-sucedido.

## METODOLOGIA

Esta é uma pesquisa de delineamento transversal, de base populacional, realizada em cidade polo do norte de Minas Gerais, Brasil. Os dados foram coletados, em 2017, entrevistando os idosos assistidos na atenção primária à saúde (APS) da área urbana. Nesse ano, o município contava com cobertura assistencial pelas equipes da ESF superior a 80%.

O tamanho da amostra baseou-se na estimativa populacional, e foi utilizada a fórmula para população infinita, com prevalência do desfecho igual a 50%, erro amostral de 3% e intervalo de confiança de 95% (IC95%). A amostragem foi complexa por conglomerados: polos regionais de saúde e equipes de ESF. Considerando-se o processo de amostragem, o número foi multiplicado por um fator de correção para o efeito do desenho (deff) igual a 1,5 e acrescido de 10% para eventuais perdas.

A equipe de entrevistadores, composta de enfermeiros e estudantes de medicina, foi especialmente treinada para a coleta de dados. Além disso, um estudo-piloto foi realizado para calibração final dos instrumentos e entrevistadores (dados não incluídos na análise final). A coleta foi domiciliar e nos períodos matutino, vespertino ou noturno, em todos os dias da semana. Os idosos ausentes em seus domicílios em pelo menos três visitas, em dias e horários diferentes, mesmo após o agendamento prévio, foram considerados perdas.

Foram utilizados dois questionários: o *Brazilian Older Americans Resources and Services Multidimensional Function Assessment Questionnaire* (BOMFAQ)^4,14^ e o Índice de Vulnerabilidade Clínico-Funcional (IVCF-20)^12,13^. O BOMFAQ é uma ferramenta multidimensional, adaptada e validada no Brasil^4,14^. O IVCF-20 foi usado para o rastreio de probabilidade ou não de vulnerabilidade clínico-funcional, com pontuação entre 0 e 40 pontos. Ele identifica os idosos frágeis com a soma maior ou igual a 15 pontos, os pré-frágeis com valor de 7 a 14 e os idosos robustos com pontuação menor ou igual a 6^12,13^. Nesse sentido, esse instrumento de *screening* reconhece idosos com menor vulnerabilidade clínico-funcional, que são provavelmente os mais ativos e com envelhecimento bem-sucedido. No presente estudo, o IVCF-20 apresentando baixa pontuação (idoso robusto) foi tomado como sinônimo de envelhecimento bem-sucedido. Assim, o escore do IVCF-20 foi dicotomizado para compor a variável dependente: menor ou igual a 6 para idoso robusto e maior ou igual a 7 para idoso não robusto.

As variáveis independentes foram compostas pelo perfil sociodemográfico (sexo, faixa etária, escolaridade, situação conjugal e renda familiar em salários mínimos da época – R$ 937,00) e determinantes do envelhecimento bem-sucedido, fundamentados no modelo tradicional de Rowe e Kahn^9^. Esse modelo, apesar de criticado, ainda influencia e é muito utilizado pela literatura^2,3,15^. Ele engloba os domínios e as respectivas variáveis avaliadas neste estudo: engajamento social (autopercepção de saúde, hábito de leitura, prática de dança e solidão), manutenção da capacidade física e cognitiva (caminhadas, prática de esportes, comprometimento cognitivo pelo Mini Exame do Estado Mental [MEEM] e sintomas depressivos pelo questionário de rastreamento psicogeriátrico [QRP]) e ausência de doenças e incapacidades (polipatologia e independência funcional avaliada por meio de atividades de vida diária [AVD]). Todas essas informações referidas acima foram obtidas a partir do BOMFAQ e dicotomizadas. Considerou-se polipatologia como cinco ou mais doenças autorrelatadas. A total independência para AVD seria o não comprometimento das atividades básicas e instrumentais, investigadas pelo instrumento BOMFAQ (deitar/levantar da cama, tomar banho, vestir-se, pentear cabelo, cortar unhas dos pés, ir ao banheiro em tempo, comer, sair de condução, subir um lance de escadas, andar perto de casa, fazer limpeza de casa, medicar-se na hora, fazer compras e preparar refeições).

Os dados foram processados pelo *software* IBM® SPSS® versão 22.0, tendo sido realizadas análises bivariadas, seguidas de análise múltipla, pela regressão de Poisson com variância robusta para todas as variáveis associadas com o evento estudado até o nível de 20% (p < 0,20). Foram mantidas no modelo final as variáveis associadas com o envelhecimento bem-sucedido até o nível de significância de 5% (p < 0,05). A análise foi efetuada para todos os idosos participantes do estudo (60 a 107 anos) e a seguir para os estratos de idosos de 60 a 79 anos (idosos jovens) e 80 anos ou mais (idosos longevos). Devido à amostragem por conglomerados, complexa, utilizou-se o peso de ponderação para estimar as razões de prevalência (RP) e IC95%.

A pesquisa foi aprovada pelo comitê de ética em pesquisa da instituição sede do estudo, mediante parecer nº 1.628.652. Os idosos foram previamente informados sobre a pesquisa e consentiram em participar, voluntariamente, assinando um termo de consentimento livre e esclarecido. O sigilo e a confidencialidade das informações coletadas foram assegurados.

## RESULTADOS

Participaram do estudo 1.750 idosos, dos quais 844 (48,7%) foram considerados “robustos”, 548 (31,2%) “pré-frágeis” e os 357 restantes (20,1%) “frágeis”. Em relação às características sociodemográficas do grupo, verificou-se que a maioria dos participantes era do sexo feminino (63,5%), alfabetizada (89,0%), tinha companheiro ou cônjuge (54,2%) e recebia até dois salários mínimos (63,5%). Idosos de 60 a 69 anos (RP = 1,15; IC95% 1,11–1,19) e 70 a 79 anos (RP = 1,09; IC95% 1,06–1,13) apresentaram maior prevalência de robustez quando comparados àqueles com 80 anos e mais, conforme apresentado na Tabela 1. A Figura apresenta a caracterização em percentuais da vulnerabilidade clínico-funcional pelo IVCF-20 dos 1.750 idosos classificados em “robustos” e “não robustos”, estratificados por idade.

**Tabela 1:** Associação entre variáveis sociodemográficas e envelhecimento bem-sucedido (regressão de Poisson) para idosos cadastrados na atenção primária à saúde em Montes Claros, MG, Brasil, 2017.

Variáveis sociodemográficas	N = 1.750 idosos n (%^a^)	Idoso robusto (escore IVCF-20 ≤ 6)				Análise bivariada		Análise múltipla	
		
		Sim		Não		p	RP (IC95%)	p	RP (IC95%)
	
		n	%^a^	n	%^a^				
Sexo						< 0,001		0,070	
Feminino	1.111 (63,5)	477	43,2	633	56,8		1		1
Masculino	639 (36,5)	367	58,2	272	41,8		1,11 (1,07–1,14)		0,98 (0,95–1,00)
Faixa etária						< 0,001		< 0,001	
80 anos ou mais	329 (18,5)	63	19,3	266	80,7		1		1
70 a 79 anos	569 (32,5)	257	45,5	312	54,5		1,17 (1,13–1,21)		1,09 (1,06–1,13)
60 a 69 anos	852 (49,0)	524	61,8	327	38,2		1,31 (1,26–1,35)		1,15 (1,11–1,19)
Alfabetizado						< 0,001			
Não	201 (11,0)	58	28,9	143	71,1		1	0,235	1
Sim	1.545 (89,0)	785	51,1	762	48,9		1,15 (1,10–1,20)		1,03 (0,99–1,06)
Situação conjugal						< 0,001			
Sem companheiro	803 (45,8)	327	41,0	476	59,0		1	0,978	1
Com companheiro	947 (54,2)	518	55,1	429	44,9		1,15 (1,10–1,21)		1,00 (0,97–1,03)
Renda familiar						0,316			
> 2 SM	1.053 (63,5)	300	50,5	298	49,5		1	-	-
Até 2 SM	568 (36,5)	500	47,9	553	52,1		1,02 (0,98–1,05)		

IVCF-20: Índice de Vulnerabilidade Clínico-Funcional; RP: razão de prevalências; IC95%: intervalo de confiança de 95%; SM: salários mínimos da época

^a^ Percentual ajustado pelo fator de correção da amostra.

**Figura:** Caracterização da vulnerabilidade clínico-funcional pelo Índice de Vulnerabilidade Clínico-Funcional (IVCF-20) de idosos estratificados por idade (60 a 107 anos, 60 a 79 anos e 80 anos ou mais) assistidos na atenção primária à saúde em Montes Claros, MG, Brasil, 2017.

Entre os determinantes do envelhecimento bem-sucedido, no engajamento social com a vida verificou-se que 71,2% dos idosos apresentavam autopercepção positiva da vida e 52,7% tinham o hábito da leitura. Nas variáveis de manutenção da capacidade física e cognição, 28,5% faziam caminhada e 88,4% não apresentavam comprometimento cognitivo. Quanto à ausência de doenças e incapacidades, 27,7% não apresentavam polipatologias e 42,8% eram totalmente independentes para as AVD. Houve associação com robustez para: autopercepção positiva da saúde, dançar, ausência de solidão, fazer caminhada, ausência de comprometimento cognitivo, não ter sintomas depressivos, não relatar cinco ou mais doenças (polipatologia) e ser independente para AVD (Tabela 2).

**Tabela 2:** Associação entre variáveis relacionadas à saúde e hábitos de vida e envelhecimento bem-sucedido (regressão de Poisson) para idosos cadastrados na atenção primária à saúde em Montes Claros, MG, Brasil, 2017.

Variáveis	N = 1.750 idosos n (%^a^)	Idoso robusto (escore IVCF-20 ≤ 6) 60 a 107 anos				Análise bivariada		Análise múltipla	
		
		Sim		Não		p	RP (IC95%)	p	RP (IC95%)
	
		n	%^a^	n	%^a^				
Engajamento social									

Autopercepção de saúde						< 0,001		< 0,001	
Negativa	511 (28,8)	105	20,7	406	79,3		1		1
Positiva	1.239 (71,2)	739	60,0	499	40,0		1,48 (1,41–1,55)		1,19 (1,13–1,24)
Hábito de leitura						< 0,001		0,690	
Não	918 (52,7)	394	43,3	524	56,3		1		1
Sim	820 (47,3)	444	54,6	376	45,4		1,11 (1,05–1,16)		1,00 (0,97–1,05)
Dançar						< 0,001		< 0,001	
Não	1.569 (90,2)	714	45,9	855	54,1		1		1
Sim	167 (9,8)	119	71,6	48	28,4		1,28 (1,19–1,38)		1,15 (1,09–1,27)
Solidão						< 0,001		0,007	
Presente	345 (19,8)	80	23,4	264	76,6		1		1
Ausente	1.381 (80,2)	763	55,6	618	44,4		1,38 (1,31–1,41)		1,07 (1,02–1,13)

Manutenção da capacidade física e cognitiva									

Fazer caminhadas						< 0,001		< 0,001	
Não	1.241 (71,5)	501	40,7	740	59,3		1		1
Sim	494 (28,5)	334	68,3	160	32,4		1,31 (1,24–1,38)		1,13 (1,08–1,18)
Prática de esportes						0,003		0,959	
Não	1.655 (95,4)	780	47,5	875	52,5		1		1
Sim	78 (4,6)	51	64,6	27	35,4		1,18 (1,06–1,32)		1,00 (0,91–1,09)
Comprometimento cognitivo						< 0,001		< 0,001	
Presente	201 (11,6)	42	21,7	159	78,3		1		1
Ausente	1.545 (88,4)	801	52,2	744	47,8		1,34 (1,25–1,43)		1,18 (1,11–1,27)
Sintomas depressivos						< 0,001		< 0,001	
Presentes	455 (25,9)	91	21,7	364	80,1		1		1
Ausentes	1.271 (74,1)	752	52,2	518	40,5		1,48 (1,41–1,55)		1,15 (1,10–1,21)

Ausência de doenças e incapacidades									

Polipatologia						< 0,001		< 0,001	
Sim	489 (27,7)	67	13,7	422	86,3		1		1
Não	1.260 (72,3)	777	62,1	483	37,9		1,67 (1,56–1,69)		1,33 (1,27–1,39)
Independência funcional para atividades de vida diária						< 0,001		< 0,001	
Não	998 (57,2)	287	29,1	711	70,9		1		1
Sim	751 (42,8)	557	74,8	194	25,2		1,56 (1,50–1,63)		1,30 (1,24–1,36)

IVCF-20: Índice de Vulnerabilidade Clínico-Funcional; RP: razão de prevalências; IC95%: intervalo de confiança de 95%

^a^ Percentual ajustado pelo fator de correção da amostra.

No grupo de idosos de 60 a 79 anos (n = 1.421), a prevalência de robustez foi 55,0%, associada às seguintes variáveis: autopercepção positiva da saúde, dançar, ausência de solidão, fazer caminhada, ausência de comprometimento cognitivo, não ter sintomas depressivos, não relatar cinco ou mais doenças (polipatologia) e ser independente para AVD (Tabela 3). Entre os idosos de 80 anos ou mais (n = 329), a prevalência de robustez foi 19,2%, associada à prática de dança, fazer caminhada, não ter comprometimento cognitivo, não relatar polipatologia e total independência para AVDs (Tabela 4).

**Tabela 3:** Associação entre variáveis relacionadas à saúde e hábitos de vida e envelhecimento bem-sucedido (regressão de Poisson) para idosos de 60 a 79 anos cadastrados na atenção primária à saúde em Montes Claros, MG, Brasil, 2017.

Variáveis	N = 1.421 idosos n (%^a^)	Idoso robusto (escore IVCF-20 ≤ 6) 60 a 79 anos				Análise bivariada		Análise múltipla	
		
		Sim		Não		p	RP (IC95%)	p	RP (IC95%)
	
		n	%^a^	n	%^a^				
Engajamento social									

Autopercepção de saúde						< 0,001		< 0,001	
Negativa	406 (28,0)	97	23,9	309	76,1		1		1
Positiva	1.015 (72,0)	684	67,6	330	32,4		1,32 (1,28–1,37)		1,14 (1,10–1,18)
Hábito de leitura						< 0,001		0,679	
Não	718 (51,0)	362	50,9	356	49,1		1		1
Sim	691 (49,0)	413	60,2	278	39,8		1,06 (1,02–1,10)		1,01 (0,92–1,04)
Dançar						< 0,001		< 0,001	
Não	1.252 (88,9)	657	52,8	595	47,2		1		1
Sim	155 (11,1)	113	73,1	42	26,9		1,15 (1,09–1,23)		1,09 (1,03–1,14)
Solidão						< 0,001		0,011	
Presente	281 (19,8)	74	26,4	208	73,6		1		1
Ausente	1.129 (80,2)	706	61,9	123	37,9		1,26 (1,22–1,31)		1,05 (1,01–1,09)

Manutenção da capacidade física e cognitiva									

Fazer caminhadas						< 0,001		< 0,001	
Não	961 (68,3)	458	47,9	503	52,1		1		1
Sim	446 (31,7)	314	71,0	132	29,0		1,18 (1,13–1,23)		1,07 (1,03–1,11)
Prática de esportes						< 0,018		0,846	
Não	1.338 (95,2)	721	54,3	617	45,7		1		1
Sim	67 (4,8)	47	69,3	20	30,7		1,11 (1,02–1,21)		1,01 (0,94–1,07)
Comprometimento cognitivo						< 0,001		0,005	
Presente	109 (7,8)	37	34,4	72	65,6		1		1
Ausente	1.310 (92,2)	743	57,1	567	42,9		1,15 (1,08–1,22)		1,08 (1,02–1,14)
Sintomas depressivos						< 0,001		< 0,001	
Presentes	357 (24,8)	83	23,1	274	76,9		1		1
Ausentes	1.054 (75,2)	697	66,4	356	33,6		1,32 (1,28–1,37)		1,11 (1,06–1,15)

Ausência de doenças e incapacidades									

Polipatologia						< 0,001		< 0,001	
Presente	366 (25,5)	64	17,4	302	82,6				
Ausente	1.054 (74,5)	717	68,4	337	31,6		1,38 (1,35–1,43)		1,21 (1,17–1,24)
Independência funcional para atividades de vida diária						< 0,001		< 0,001	
Não	743 (52,5)	260	35,3	483	64,7		1		1
Sim	677 (47,5)	521	77,4	156	22,6		1,33 (1,29–1,39)		1,18 (1,14–1,22)

IVCF-20: Índice de Vulnerabilidade Clínico-Funcional; RP: razão de prevalências; IC95%: intervalo de confiança de 95%

^a^ Percentual ajustado pelo fator de correção da amostra

**Tabela 4:** Associação entre variáveis relacionadas à saúde e hábitos de vida e envelhecimento bem-sucedido (regressão de Poisson) para idosos de 80 anos ou mais cadastrados na atenção primária à saúde em Montes Claros, MG, Brasil, 2017.

Variáveis	N = 329 idosos n (%^a^)	Idoso robusto (escore IVCF-20 ≤ 6) 80 anos ou mais				Análise bivariada		Análise múltipla	
		
		Sim		Não		p	RP (IC95%)	p	RP (IC95%)
	
		n	%^a^	n	%^a^				
Engajamento social									

Autopercepção de saúde						< 0,001		0,284	
Negativa	105 (52,5)	08	8,3	97	91,7		1		1
Positiva	224 (67,7)	55	24,5	169	75,5		1,10 (1,05–1,15)		1,04 (0,97–1,13)
Hábito de leitura						0,135		0,828	
Não	200 (60,4)	32	16,0	168	84,0		1		1
Sim	129 (39,6)	31	24,2	98	75,8		1,04 (0,99–1,10)		0,99 (0,91–1,07)
Dançar						0,045		0,035	
Não	317 (96,4)	57	18,1	260	81,9		1		1
Sim	12 (3,6)	06	50,0	06	50,0		1,22 (1,02–1,48)		1,32 (1,02–1,71)
Solidão						0,006		0,371	
Presente	63 (19,7)	06	9,8	57	90,2		1		1
Ausente	252 (80,3)	57	22,7	195	77,3		1,07 (1,02–1,13)		0,97 (0,89–1,04)

Manutenção da capacidade física e cognitiva									

Fazer caminhadas						< 0,001		0,026	
Não	280 (85,5)	43	15,5	237	84,5		1		1
Sim	48 (14,5)	20	41,9	28	58,2		1,17 (1,06–1,28)		1,16 (1,02–1,32)
Prática de esportes						0,243		-	
Não	317 (96,4)	59	18,7	258	81,3		1		-
Sim	11 (3,6)	04	38,2	07	61,8		1,11 (0,93–1,34)		-
Comprometimento cognitivo						< 0,001		0,004	
Presente	92 (96,4)	59	18,7	258	81,3		1		1
Ausente	11 (3,6)	04	38,2	07	61,8		1,10 (1,05–1,15)		1,11 (1,03–1,20)
Sintomas depressivos						< 0,001		0,557	
Presentes	98 (30,5)	08	8,1	74,5	91,9		1		1
Ausentes	217 (69,5)	55	25,5	162	74,5		1,10 (1,05–1,15)		1,02 (0,95–1,01)

Ausência de doenças e incapacidades									

Polipatologia						< 0,001		< 0,001	
Sim	123 (37,3)	03	2,7	120	97,3		1		1
Não	206 (62,7)	60	29,1	146	70,9		1,16 (1,11–1,21)		1,24 (1,16–1,33)
Independência funcional para atividades de vida diária						< 0,001		< 0,001	
Não	255 (77,8)	27	10,7	228	89,3		1		1
Sim	74 (22,2)	36	49,5	38	50,5		1,26 (1,16–1,36)		1,33 (1,18–1,51)

IVCF-20: Índice de Vulnerabilidade Clínico-Funcional; RP: razão de prevalências; IC95%: intervalo de confiança de 95%

^a^ Percentual ajustado pelo fator de correção da amostra

## DISCUSSÃO

Entre os idosos assistidos pelas equipes da ESF, na APS, a prevalência de robustez pode ser considerada satisfatória, quando avaliada entre todos os idosos participantes do estudo. Aproximadamente metade dos idosos foi estratificada em baixa vulnerabilidade clínico-funcional, ou seja, potencialmente ativa e independente. Outros estudos apresentaram percentual menor de idosos robustos, como os de Hank^19^(8,5%), McLaughlin^15^(10,9%), Curcio^18^(24,4%), Canedo^2^(25%) e Bosch-Farre^20^(23,5% ou 38,9%, conforme instrumento utilizado). Na análise ajustada por idade, verificou-se entre idosos de 60 a 79 anos uma prevalência de robustez quase três vezes maior em relação àquela encontrada nos indivíduos de 80 anos ou mais, observação similar em estudo no Rio de Janeiro^2^. Nos três grupos de análise (todos os idosos, 60 a 79 anos e 80 anos ou mais) foram associadas à robustez as seguintes variáveis: prática de dança, fazer caminhada, ausência de comprometimento cognitivo, não relatar polipatologia e total independência para AVD.

Todavia, é importante considerar o fato de que não existe padronização de instrumentos para aferir o envelhecimento bem-sucedido. De forma similar, a categorização para as faixas etárias é diferente entre os estudos, assim como as metodologias utilizadas. A proposta mais clássica de Rowe e Kahn^9^, apesar das ponderações presentes na literatura, continua influenciando significativamente todas as discussões desse assunto^15^. Estudos sobre o tema são promissores, porém não existe consenso conceitual ou instrumentos padronizados universalmente para a avaliação^5,15,17,19^.

O processo de envelhecimento é desafiador e exige modelos inovadores de atenção à saúde, isto é, capazes de identificar e acompanhar as condições clínico-funcionais da pessoa idosa de forma rápida, precoce e contínua, particularmente na rede pública de saúde^1,5,10,11^. Atualmente, a abordagem da saúde da pessoa idosa deve alicerçar-se na interação da funcionalidade do indivíduo (autonomia e independência) com o seu ambiente^1,5^. Dessa forma, na literatura iniciam-se reflexões sobre a evolução positiva, multidimensional e integrada que constitui o processo de envelhecimento^1,2,5,11,17,18,20^.

O IVCF-20, utilizado no presente estudo, foi desenvolvido para a estratificação do risco clínico-funcional e pode ser considerado indicador de boas condições de saúde, de capacidade da saúde ou da funcionalidade global^12,13^. Ele permite, além de classificar os idosos com elevada e moderada vulnerabilidade funcional, identificar aqueles considerados de baixo risco clínico-funcional, isto é, os robustos. Os indivíduos identificados com o IVCF-20 menor que sete pontos apresentam-se mais saudáveis e ativos, e devem manter o acompanhamento habitual com o foco nas medidas de prevenção e promoção da saúde na atenção primária^13^. Ela é a porta de entrada para a rede de atenção à saúde e atua como coordenadora do cuidado, e portanto precisa ser capaz de integrar outros pontos de atenção com maior complexidade, de acordo as condições clínico-funcionais da população idosa^10,11^.

Nesta investigação, na análise de todos os idosos, a idade foi a variável sociodemográfica significante no modelo final. A redução da prevalência de robustez entre idosos com 80 anos ou mais ficou evidenciada no atual estudo. Outros trabalhos, apesar de utilizar instrumentos diferentes, mas com critérios semelhantes, também demonstraram que idosos mais jovens apresentam-se mais saudáveis e robustos^2,10,18,20^. Contudo, o envelhecer contempla questões multidimensionais^7,8^ com envolvimento de diferentes preditores, que são influenciados no curso da vida^1,11,16^. Idosos mais jovens e independentes, em ambientes favoráveis, apresentam melhor percepção da vida e são mais ativos que os longevos^2^. Neste estudo, não se queixar de “solidão” foi associado à robustez no grupo dos idosos em geral e naqueles de 60 a 79 anos. Portanto, a interação entre independência funcional e ambiente favorável promove satisfação e êxito no engajamento ativo ao longo da vida^1,2,21^. Os estudos com idosos de 80 anos ou mais são escassos e com metodologias limitadas, carecendo de investigações^2,22^ quanto ao engajamento social.

O envelhecimento bem-sucedido pode ser reproduzido na capacidade funcional por habilidades físicas e mentais, essenciais na autonomia e independência de cada pessoa em ambiente amistoso (físico e social). Isso é indispensável para o bem-estar de todo ser humano, no sentido mais amplo, incluindo os domínios felicidade, satisfação e autoeficácia^1,2^. Nesta investigação, os idosos com autopercepção positiva da vida e que praticavam a dança como entretenimento apresentaram menor vulnerabilidade clínico-funcional, provavelmente por desenvolverem trajetórias bem-sucedidas no envelhecer e vice-versa, com destaque para a variável “prática de dança” associada à robustez entre idosos longevos (80 anos ou mais) e mais jovens (60 a 79 anos). A literatura também demonstrou que as pessoas idosas capazes de gerenciar a própria vida (autonomia) e realizar atividades de lazer revelaram uma autopercepção da vida otimista, o que contribui, de forma particular, para uma velhice saudável e ativa^1,2,23^.

Para além daqueles idosos com envelhecimento bem-sucedido, deve-se também enfatizar os resultados do atual trabalho quanto à prevalência de não robustez, que atinge especialmente a grande maioria dos idosos longevos. Portanto, faz-se necessário também investir na capacitação de profissionais de saúde quanto à estratificação clínico-funcional e cuidado centrado nas particularidades dos idosos pré-frágeis e frágeis. Nesse contexto, a qualificação profissional da equipe, no âmbito da saúde pública, poderia contribuir para efetivar medidas de recuperação da saúde e reabilitação da funcionalidade dos indivíduos vulneráveis. Também é importante que gestores propiciem ambientes públicos, estruturalmente saudáveis, para essa população.

A perspectiva intersetorial do envelhecer saudável e ativo, em ambientes amigáveis, pode oportunizar tanto a manutenção quanto a restauração da capacidade física e cognitiva^1,11,21,27^. Ademais, a OMS, desde 2007, por meio do guia global das Cidades Amigas dos Idosos, já recomendava o ambiente amigável para essa população. O guia sugere adaptação das estruturas e integração entre os sistemas para promoção de um envelhecimento bem-sucedido e ativo^27^. Na atualidade, o documento Brasil Amigo da Pessoa Idosa reforça essa proposta anterior e assume compromissos com municípios que aderirem e cumprirem os requisitos determinados pela iniciativa^28^. Essa estratégia, em conformidade com o novo cenário epidemiológico e social da população brasileira, pode colaborar no enfretamento dos desafios frente ao envelhecimento, repercutindo de maneira benéfica na capacidade clínico-funcional.

Outro ponto significante relacionado à longevidade saudável foi que os idosos com capacidade cognitiva e independentes funcionalmente adquirem vários comportamentos saudáveis ao longo da vida^2,24,26,29^ e inclusive podem usufruir de tecnologia digital no gerenciamento da saúde^30^. Essa afirmação reitera os achados desta pesquisa, na qual os entrevistados considerados robustos apresentaram maior prevalência de ausência de comprometimento da cognição ou sintomas depressivos e realização de caminhadas. Portanto, é de fundamental importância estabelecer estratégias que mantenham esses idosos altamente funcionais, pelo maior tempo possível, na progressão da sua existência. Isso concorre para uma velhice bem-sucedida e de qualidade^20,31^, com menores taxas de morbidade e mortalidade^31^.

A ausência de incapacidades e doenças compõe outro grupo de fatores determinantes para o envelhecimento bem-sucedido^9^. Nesta pesquisa, os idosos que se apresentavam sem relato de polipatologias e com total independência para todas as AVD tinham superioridade na capacidade clínico-funcional em relação aos seus pares. Esses achados foram encontrados também para a análise estratificada por idade. Outros estudos também demonstraram o quanto a presença de incapacidades e polipatologias produz vulnerabilidade clínico-funcional nos indivíduos, com impactos negativos na saúde e no percurso da vida^1,2,4,10,18,31^.

Os resultados do presente estudo devem ser considerados à luz de algumas limitações. É um trabalho transversal que impossibilita a determinação da causalidade. Os dados foram relatados pelos idosos, devendo-se levar em conta o viés de memória. Além disso, os instrumentos de coleta de dados possuem limitações, apesar de permitirem estratificar os indivíduos em suas características de saúde. Nessa perspectiva, deve-se considerar a importância de distinguir e referenciar os idosos “frágeis” para avaliação clínica multidimensional e elaboração do plano de cuidados, no nível secundário da rede de atenção pública, com respectiva contrarreferência para acompanhamento longitudinal pela equipe de saúde da família. Os indivíduos em fragilização e os robustos prosseguem com a assistência pelos profissionais da APS capacitados nas particularidades da saúde do idoso, de acordo com orientações dos manuais e/ou diretrizes de atenção à saúde.

Apesar das limitações apresentadas, o desenho amostral e o elevado número de idosos incluídos garante representatividade do grupo estudado. O instrumento IVCF-20 é um questionário de rastreio, que permite a estratificação clínico-funcional dos idosos. Ele é validado e de fácil aplicação, podendo ser usado por qualquer profissional da saúde, facilitando a triagem inicial e o acompanhamento dessa população pela ESF.

Em síntese, no presente estudo evidenciou-se importante prevalência de idosos ativos e saudáveis (robustos), isto é, de pessoas consideradas com baixa vulnerabilidade clínico-funcional. No entanto, a análise ajustada para idosos longevos demonstrou significante redução nessa prevalência, resultado que reforça a urgência em redesenhar o sistema de atenção à saúde do idoso, com foco especial para as particularidades das diferentes faixas etárias, de forma a prolongar o tempo de vida com engajamento ativo e livre de incapacidades físicas ou cognitivas. Ressalta-se, portanto, a necessidade de educação permanente dos profissionais no cuidado da pessoa idosa, com promoção da saúde e prevenção da vulnerabilidade clínico-funcional, protelando o desenvolvimento das enfermidades e suas complicações, além de capacitação da equipe da APS para ações de recuperação da saúde e reabilitação da funcionalidade.

Neste contexto, muitos desafios estão lançados. Recomendam-se novas pesquisas nesta temática com o propósito de estimular o estudo da relação entre determinantes do envelhecimento bem-sucedido e idosos com baixa vulnerabilidade clínico-funcional (robustez), além de avaliações sobre o planejamento e a execução das políticas públicas para esse contingente populacional.
